# Diseases of the Maxillary Sinus

**Published:** 1856-07

**Authors:** J. A. Giraldes, Christopher Johnston

**Affiliations:** Professor agrege a la Faculte de Medecine, &c. &c., Paris.; Baltimore.


### ARTICLE VII.

*Diseases of the Maxillary Sinus.* By DR. J. A. GIRALDES, Professor agrege a la Faculte de Medecine, &c. &c., Paris. Translated by Dr. CHRISTOPHER JOHNSTON, Baltimore.

*Anatomical Considerations.*—The superior maxillary bones constitute the principal part of the facial skeleton; their form, their volume contribute to give the physiognomy the particular traits which characterize different races, and impress upon the physiognomy of individuals those numerous peculiarities which are met with in the same family at different epochs of life, in the child, the adult and the aged. The modifications which belong to epochs result especially from the transformations through which the superior maxillary bones pass before reaching their perfect development. At birth and in early life, the superior maxillary bone is hardly developed, but it contains within it important organs which, during their own perfecting, give it a new form; it consists of a spongy tissue, of which the meshes are eventually pushed back to lodge a mucous sac, a dependence or process of the Schneiderian membrane, destined ultimately to line a great cavity—the MAXILLARY SINUS. There is, consequently, a difference between the superior maxillary bone of the child and of the adult; this difference, besides other peculiarities, consists in the presence of a cavity—a sinus—which increases the volume of the bone without augmenting its weight—a sinus still known as the antrum of Highmore.

We will now examine this sinus rapidly, and especially in a surgical point of view.

The antrum of Highmore, in opposition to the belief of some, was not discovered by the anatomist whose name it bears; others before him had noticed it, but Highmore*

* Highmore, corporis humani disc. anatomica Hagæ, 1651, p. 226, tab. xvi.

has the merit of having best described it, and above all of having best established its relations with the roots of the molar teeth. And especially is it since he put forth his description of the sinus, made more conspicuous by means of an enlarged but exact figure, that the importance of this anatomical point has been fully appreciated; and it is only since that time that various diseases of the antrum of Highmore have become known to us.

The maxillary sinus occupies almost entirely the superior maxillary bone, and is hollowed out of that part of the os maxillare which is independent of the incisor teeth—it constitutes a great cavity of quadrangular form, having the figure of a quadrangular pyramid: the base corresponds to the nasal fossæ, and the angles and edges are rounded correspondingly to points of the maxillary bone, which deserve mention. The superior face answers to the floor of the orbit, and the anterior to that point of the bone known as the canine fossa; at this spot the weakness of the wall allows of easy penetration into the sinus. The external face looks a little backwards. The inferior, rounded border is in direct relation with the roots of the molar teeth which sometimes penetrate into the cavity of the sinus, being separated from it by a thin, porous lamella only, which offers no obstacle to the operator. The anterior and inner angle may extend even into the base of the ascending process; the external angle corresponds to the molar process, and occasionally penetrates it. The dimensions of this cavity vary with the individual, and especially according to age; sometimes its walls are depressed, pressed inwards, and then its cavity is narrowed. The sinus may even completely disappear, as in a case cited by Morgagni.* In some instances, partitions divide the cavity into several compartments; but a more important point, one which did not escape Highmore, is the variable thickness of the walls, which not unfrequently are constituted solely of thin lamellæ very easily broken.

* Advert. anat.

The cavity of the sinus communicates directly with the nasal fossæ. It is lined by the Schneiderian membrane, which, in penetrating into the antrum, becomes thinner without losing any of its characters. Like that of Schneider, it rests upon periosteum attached to it by an easily demonstrable areolar tissue. This periosteum is provided with nerves derived from the dental nerves, which enter its tissue and are therein distributed, whilst other branches lie in contact with it; these nerves proceed from the fifth pair.

The membrane of the maxillary sinus offers differences in thickness in different points; it is thicker on the inner surface—that in relation with the nasal fossæ—than elsewhere; it is more vascular there and is richer in mucous glands. Besides, all the surface of the membrane is covered with a cylindrical epithelium.

The cavity of the sinus communicates with that of the nasal fossæ by an opening the size of a crow's quill. This aperture, sometimes double, is placed at the upper part of the middle meatus, overhung by the corresponding turbinated bone, and it ends in a depression common to the ethmoidal and frontal sinuses. It is easy to perceive that owing to varieties in the volume and form of the turbinated bone, catheterism of the maxillary sinus may be oftentimes difficult; and that in attempting to traverse the opening the wall may be perforated, a fact which has been established by the commission of the academy of surgery.

The maxillary sinus presents still other varieties depending upon the different phases of dental evolution. Before the development of the last molars its posterior and inferior part is occupied by the bulb, and the periosteum of the sinus is in intimate relation with these organs, which explains some of the disturbances propagated by continuity of tissue in the cavity of the sinus, in consequence of impediments which the dental organ finds opposed to its normal development. Further, in many cases abnormal teeth are met with in this cavity, and they produce diseases of which we will presently speak: and finally, the attentive examination of the anatomical disposition of the maxillary sinus, of its volume, of its relations, informs us how diseases of neighboring parts may invade it, obliterate its cavity, and suggest the existence of an affection developed in its interior.

Chapter II.—*Pathology.*—The maxillary sinus is a cavity with osseous walls covered with a periosteum, and lined with mucous membrane; the diseases of which it is the seat ought necessarily to originate in one of these three parts—the bone, the periosteum, and the mucous membrane. But this distinction, sufficiently correct in an anatomical point of view, cannot be maintained in a clinical survey; wherefore, we will not pursue it, adopting in preference, for the following study, the physiological order.

The maxillary sinus performs functions of which we have nothing to say; but there take place in its tissues phenomena of nutrition, the disturbance of which may provoke the development of lesions, which are the diseases observed in it. It may also, like any other part of the body, be the object of external violence. The diseases to which it is liable are consequently of two orders; the first are produced by external violence; the others on the contrary are the result of a modification of nutrition, involving exudation of plastic fibrinous matter susceptible of being converted either into *pus* or new tissues. We divide, then, our subject into two parts: 1st. Traumatic lesions; 2d. Diseases produced by an alteration or perversion of nutrition, the latter determining the products which I have just pointed out.

The first group comprehends fractures and contusions. In the second we place inflammation, its sequelæ, and the tumors or accidental products developed from a local cause or a general condition of the organism.

Section I.—*Traumatic Lesions.*—Under the title traumatic lesions I include every lesion produced by a mechanical cause; these are contusions, fractures, effusions of blood. Finally, in a concluding paragraph, I will treat of foreign bodies that may be met with in this cavity, having been introduced into it by external violence.

1st. *Contusions and Fractures.*—Fractures of the maxillary sinus are almost always accompanied with contusion of the surrounding parts, and ought not to constitute a distinct chapter. For instance, when the anterior part of the face receives a violent blow, it seldom happens that the surface corresponding to the sinus is alone crushed; almost always the lesions extend more or less on every side. Nevertheless, in rare cases the sinus may be directly contused and beaten in. A thrust of a foil, of a bayonet or of a sabre, sometimes produces this result. Beclard relates the history of a man who had received a blow from an umbrella stick in this region; the ferule of the umbrella fractured the bony walls of the sinus, and, becoming detached, lodged in the cavity.

In other instances the lesions are spread over a more extended surface. Brodie* cites the case of a child which, having received a violent blow upon the cheek, presented a tumor in this region, and eventually necrosis around the sinus.

2d. *Effusions of Blood.*—Contusions of the anterior part of the cheek may, in some instances, give rise to effusion of blood in the sinus. Dupuytren† received in his wards a young girl, who entered the hospital for a tumor of the face resulting from a blow. An augmentation of volume was observed in the region; the eye projected out of the orbit;—two days afterward Dupuytren plunged a bistoury into the tumor, whereupon a certain quantity of blackish blood escaped at the incision. The finger pressed into the cavity of the sinus encountered a soft and lacerable mass; and the next day the surgeon, widening the opening, withdrew two ounces of blood. Jourdain‡ relates that a young lady, fifteen years of age, had an effusion of blood

* London Med. Gazette, vol. xv, p. 346.

† Dupuytren, cl. chir. t. iii.

‡ Recherches sur les mal. du Sinus Max. Jour. de Med., 1757, tab. ii, obs. v.

into the sinus in consequence of fracture of the dental arch. Knorz* speaks of having met with a sanguineous effusion at the same point, in a man who died after a fall from a great elevation. These facts, and another instance observed in 1847 in the wards of Prof. Velpeau,† afford evidence that sanguineous effusions really take place in the maxillary sinus; that the blood there undergoes some of the modifications noticeable in effusions elsewhere, that it may coagulate or even pass into decomposition.

Sanguineous effusions in the antrum may sometimes give rise to inflammation in that cavity; this condition is evidenced by pain in the upper jaw, and by formation of a tumor in the region of the cheek extending at times to the orbitar side. It is of consequence to be acquainted with the fact that these effusions may have added to them the matters secreted in the cavity and the serosity poured out by an irritated mucous membrane. Under the supposition that the tumor is malignant, the surgeon will verify his diagnosis by means of an explorative puncture; he will thus assure himself of the presence or absence of a liquid and of its nature. If any exist, he may also employ the means indicated by M. Bermond,‡ a means not recommended by experience. According to this surgeon, whenever the sinus is filled with fluid, the presence of the latter may be ascertained by auscultation of the jaw. In the normal condition, says this author, the passage of air into the cavities of the maxillary sinuses occasions a *bruit*—a sound—which is absent when these are filled with fluid.

The *treatment* of fractures and contusions is comprised in the study of fractures of the bones of the face. It consists in combating existing complications, and in abstaining, even in comminuted fractures, from extracting the fragments, unless they produce disturbance.

* Knorz, De maxillæ superioris in primis ejus sinus morbosis affectionibus. Marb., 1844.

† Gazette der Hopitaux, 1847, p. 289.

‡ Gazette Med. de Paris, t. ix, p. 253.

When blood is effused into the sinus, especially when its presence provokes inflammation, it is indispensable to give it exit. For this purpose a perforation may be made in the sinus, or else the extraction of one or two carious teeth may allow of penetration through the alveoli, and of removal of the liquid by means of injections.

3d. *Foreign bodies in the Sinus.*—Foreign bodies met with in the maxillary sinuses have always entered these cavities by traumatic lesion; they are fragments of iron, of projectiles urged by gunpowder, of stones, &c.

Ravaton* relates the history of a Swiss sergeant who, at the battle of Rosbach, received a bullet under the orbit. The wound remained fistulous a long time, but the ball was at length discovered. Dupuytren tells us of an officer into whose sinus a ball had penetrated. In Beclard's case also, the umbrella ferule was retained for a considerable period.

The presence of foreign bodies in this cavity maintains suppuration and renders fistulous the wounds by which they obtained access; and through the fistula they may generally be recognized with a probe. In the memoir of Bordenave occurs the history of a young man bearing a fistula in the cheek maintained by a plug of lint. Allonel† cites the observation of a gun-shot wound of the cheek made fistulous by a morsel of shell. We must also regard as foreign bodies the lachrymal cannulas inadvertently introduced into the cavity of the sinus. Finally, some authors, Pallas‡ amongst them, speak of the presence of worms, lumbrici, &c., in the maxillary sinus; but observations relating to facts of this nature are not sufficiently detailed, and are in some instances unreliable.

Foreign bodies lodged in the antrum ought to be removed—their presence not only rendering wounds fistulous, but also giving rise to erysipelatous disturbance of more or less gravity.

* Chirurgien d'Armee, 1767, obs. 31, p. 181.

† Mem. de l'Acad. Roy. de Chir. t. v.

‡ De insectis viventibus intra viventia.

SECTION II.—*Inflammation.*—It is only since Nathaniel Highmore described the maxillary sinus, that its diseases have been studied—diseases which before his time were confounded under the denominations *epulis*, *ozœna* and *polypus*, for in looking over ancient authors, it is easy to recognize by these appellations the principal features of affections developed in the maxillary sinus. In the last century only did surgeons advantageously study diseases of the antra; and Ringue* and Jourdain† especially invite attention to the subject. Subsequently Bordenave‡ includes in two memoirs read before the Royal Academy of Surgery the most important matters in connection with diseases of the maxillary sinuses. But above all, to the impulse given to pathological anatomy on the one hand, and to the advance of operative medicine during this century on the other, are we indebted for a clearer acquaintance with diseases of these cavities. Surgery now opposes an important operation against the invasion of tumors, and this is amputation or resection of the superior maxillary bone, as conceived and practiced by Lizars || and Gensoul.§

However, since surgery was enriched with this important discovery, the pathology of the sinus has remained stationary, and a regretable confusion still reigns in the study of diseases seated in this cavity. We even venture to assert that the pathology of the sinus yet remains to be made out, and that the morbid products therein developed call for fresh examination with and without the *microscope;* and it is only when these studies shall have been made that a distinction may be established between the now confused abnormal products, that the gravity of all these affections will be appreciated, and that the proposed operations may be safely adopted or wisely rejected.

* Haller. disputationis chirurg. de morbis præcipui sinuum, t. i, dis. xi.

† Journal de Med., 1767. ‡ Mem. de l'Acad. chir. obs. iv, et v.

|| Anatomical plates, t. ii, part ix, 1826. § Lettre chirurgicale.

Section I.—*Inflammation of the mucous membrane of the Sinus.*—Inflammation of the mucous lining of the maxillary sinus may be either acute or chronic. It occurs after fractures, wounds, blows upon the cheek or the teeth, &c.; but it is most frequently met with as a phenomenon of contiguity accompanying inflammation of the alveolar periosteum, complicating dental caries. Some authors, Jourdain and Brodie* amongst others, are of opinion that inflammation of the membrane of the sinus often depends upon an internal cause, such even as rheumatism. Jourdain endeavors to show that in many cases this inflammation ought not to be referred to the presence of a carious tooth. Rust† admits that internal causes most frequently give rise to this inflammation. He even distinguishes inflammation of the fibrous membrane of the periosteum, from that affecting the internal or mucous membrane; he entitles the one *gengantritis* and the other *eiagantritis*, and he refers both to general causes.

Inflammation of the maxillary sinus manifests itself by local and sympathetic symptoms, and by general symptoms.

*Local Symptoms.*—A lively and intense pain, deep in the jaw, extending from the side of the head and the ear, accompanied with great sensibility of the eye, lachrymation, ringing in the ears, great tenderness at the roots of the teeth, becoming acute pain upon the slightest touch, together with swelling of the face and persistence of the pain.

*General Symptoms.*—Fever, a state of irritability, nervous exaltation, sometimes vomiting and occasionally delirium.

*Diagnosis.*—Inflammation of the mucous membrane of the sinus frequently coincides with inflammation produced by a carious tooth; indeed we often meet with all the symptoms just enumerated resulting from the latter cause. But it deserves mention that when the inflammation is provoked by a carious tooth, by inflammation of the dental periosteum, the pain, after a certain time, diminishes

* London Med. Gazette. † Encyclop. Wærterbuch der Med. Niss., t. iii.

instead of increasing. In inflammation of the membrane of the sinus, on the contrary, the pain is more persistent, more intense and more vexatiously enduring.

Inflammation of the sinus may terminate in resolution and suppuration. When it follows after dental caries it is probably a concomitant symptom, and ends in resolution.

When suppuration is once established, an abscess or purulent accumulation is formed in the sinus, and the walls dilating, a tumor appears upon the cheek. Jourdain* gives the case of a nun whose maxillary sinus presented this condition for a year, and was accompanied with œdema of the face. Beaupreau† tells us of a woman aged twenty-five years whose antrum was distended with pus. M.Cloquet‡ saw in the Clinical Hospital a patient aged thirty years in whom the maxillary sinus formed a tumor in the cheek as large as a hen's egg. Dilatation of the sinus, in some instances, occurs not only externally but also towards the orbit. Boeneck§ records an observation of a woman aged sixty years, in whom this condition obtained.

Purulent accumulation in the maxillary sinus keeps up an irritation there; the pus mingling with mucus increases the quantity of fluid contained in the sinus, which undergoes considerable dilatation, as in the case of Boeneck.

When the abscess is formed there follows a process of erosion; the tumor pierces the osseous walls; it finds an issue either towards the mouth or else the nasal fossæ. The purulent matter irritates the mucous membrane, rendering it thick and spongy, as in Beaupreau's case, and in another instanced by Gooch, and the suppuration is maintained.

The osseous walls of the antrum may be affected;—they are oftentimes carious. Gooch‖ observed an abscess of the maxillary sinus of three years' standing which had occasioned caries of the bone. In these cases fistulæ are

* Loc. cit. † Mem. de Bordenave. ‡ Lancette Francaise, t. x, p. 82
§ Gazette Med. ‖ Caries in Surgery, vol. ii, p. 63.

formed, and are kept up by the carious condition of the osseous walls of the sinus. Observations of this kind are met with in the work of Jourdain and in the memoir of Bordenave.

Let me remark, however, that abscess of the antrum may continue for a long time without perforation, as appears by the case referred to of Gooch.*

*Progress and Diagnosis.*—When abscess forms after inflammation of the lining membrane of the sinus, its existence is recognized by a tumor in the cheek which has been preceded by sharp pain, persistent and intense. This tumor increases, distends the sinus and occasions phenomena of compression, especially towards the orbit. When the abscess is of some standing, when, more particularly, it formed without antecedent sharp pains, as in the case of Cloquet, the distinction becomes more difficult, and the abscess might be mistaken for dropsy of the maxillary sinus, unless an issue of pus through the nares, or an explorative puncture, assist the diagnosis.

*Treatment.*—The treatment of inflammation of the maxillary sinus is either local or general. Whenever we can satisfy ourselves that the disease of the sinus is not dependent upon a general or specific cause, antiphlogistic treatment ought to be employed. Leeches in numbers upon the cheek or the gengival mucous membrane; cataplasms, fomentations and emollient fumigations may be used with advantage. The general treatment consists in diet, purgatives, calomel in doses of 4 or 6 grains; and besides, an especial medication is required when the phlogosis depends upon a *specific cause.*

When the inflammation ends in suppuration—in abscess—when, for instance, fistulæ exist at the level of the alveolar arch, &c., what course must the surgeon pursue? When abscess is recognized, must it be opened, and at what point should the opening be made? Does the ab-

* Medical Times, 24th August, 1850.

scess project at some particular spot? Is it accompanied with dental caries? In the first case a free opening should be made in the most depending portion; afterwards injections are to be employed to expel all residual purulent matter; and finally, iodureted water is advantageously injected into the sinus in order to effect a modification of the mucous membrane of the cavity.

In the second case, that is to say, when the teeth are carious, it is highly advantageous to remove the diseased teeth, to perforate the bottom of the alveolus, and to widen the aperture (as did Meibomius) to admit the passage of the injections already mentioned. Jourdain insists upon the employment of injections made through the nasal fossæ; but, as we have already remarked, catheterism of the maxillary sinuses is fraught with difficulty; and it was ascertained by the Commission of the Academy of Surgery* that in the majority of cases, the catheter had perforated the wall of the nasal fossæ. It is an incontestable fact that the use of injections in purulent collections of this region, as elsewhere, gives excellent results. We will remark casually that M. Hullihen† claims to have cured *tic douloureux* of the face by means of injections of nitrate of silver thrown into the maxillary fossæ.

Abscesses of the antrum of Highmore which open spontaneously often occasion fistulæ, and these are kept up either by the condition of the bone, of the mucous membrane, or by their elevated position. When such fistulæ exist it becomes necessary to ascertain the true state of the sinus, of the bone and of the teeth. It is also requisite to explore the tract of the fistulæ and the diseased cavity; and, if necessary, to make a puncture at a more depending point. If caries exist, iodureted or other injections should be used; if necrosis be present, the sequestra should be extracted. But necrosis, be it observed, is less frequently a sequel of abscess than a result of disease of the max-

* Henr. Meib. de abscessum internorum natura et dissert., 1718.

† Hullihen, Charleston Med. Journal, 1839, vol. iv, July No. 4, p. 50.

illary bone, and usually follows contusions and affections of the cheek. Baron* describes an affection of the cheek in children in which the superior maxillary bones were necrosed. The instance cited by Lassus of "necrosis of the sinus" is rather necrosis of the superior maxillary. The same may be said of those necroses produced by phosphureted vapors, and described with much care by Bibra, Geist† and M. Heyfelder.‡

SECTION III.—*Dropsy of the Maxillary Sinus.*—Under the denomination, dropsy of the maxillary sinus, Deschamps (the son) described a morbid condition of the sinus, characterized by dilatation from an inclosed accumulation of a white or slightly yellowish glairy fluid.

This dilatation is always accompanied with thinning of the osseous parietes of the cavity. Dilatation and accumulation of a non-purulent liquid are the principal features of the disease; and these characters, as will presently be seen, serve to distinguish it from another affection with which it might be confounded, namely cysts developed in the thickness of the jaw. But before Deschamps drew the attention of surgeons to this disease, Runge had already given to the world an observation of dropsy of the maxillary sinus.

If we examine the maxillary sinuses of a great number of crania, we encounter very important peculiarities which explain the varieties of form assumed by this dropsy. Often the cavities are dilated, sometimes in their whole extent, and their thinned and transparent walls are easily depressed with the finger, but they return to shape with a characteristic noise; or else, on the contrary, they are as brittle as mica. In other instances the dilatation is directed outwards; it then effaces that bevel running from the molar apophysis, and there forms a swelling. In other cases, again, it is the anterior part of the sinus which un-

* Bulletin de la Faculte de Med., vol. v, p. 151.

† Die Krankheiten in den Phosphorzundholzfabriken, Erlang, 1847.

‡ Medic. lit.

dergoes dilatation; it is now prolonged into the ascending or nasal process of the superior maxillary bone, and forms, in the dry bone, a rounded tumor of variable size. Sometimes the upper and posterior parts of the sinus are alone affected; and there exists behind the orbit a tumor which constricts the maxillary fissures as well as the apex of the orbitar cavity. Finally, in some instances, the dilatation directs its course backwards towards the molar tuberosity, and extends, not unfrequently, into the thickness of the dental arch.

Dropsy of the maxillary sinus is thus manifested: Delavacherie* records an observation of a man thirty-six years of age, in whom the dilatation of the sinus was continued into the nasal fossæ and the palate, and caused a twisting or deformity of the wings of the nose. M. Bertrand† noticed a dropsy of the sinus in a man aged thirty-seven years; the dilatation of the cavity extended as well into the mouth as the nose, the nasal process being much distended. In the two cases I have mentioned the osseous walls had become extremely thin, and yielded to pressure; indeed, they allowed the finger applied against one side of the tumor to receive the pressure of another finger compressing a distant point of it.

The nature of the liquid contained in the sinus is subject to variation; sometimes it is thick and ropy, and sometimes it is less tenacious and slightly colored. The chemical composition of this fluid is not given; but we may state that in a case observed by Ferguson‡ it contained cholesterine. The difference in tenacity, consistence and color depends upon undetermined causes.

*Causes.*—It has been supposed that these dropsies were owing to stricture or obliteration of the communicating canal between the sinus and the nasal cavities, the accumulation of the liquid in the sinus being thereby facilitated;—this is pure hypothesis, and no observation has been

* Memoire sur quelques Maladies de la Machoire, Bruxelles, 1843.

† Lombard, These de Montpellier, 1836. ‡ Medical Times, May 1850, p. 368.

adduced to prove it. The cause assigned by T. Bell* for the formation of these fluid collections is more plausible. According to this author, they depend upon a modification of the mucous lining membrane: a modification brought about by the process going on in the roots of the teeth. In some instances the existence of serous collections in the sinus was coincident upon the presence of abnormal teeth in its cavity. The facts noticed by Dubois and Gensoul,† of incisor teeth formed in the cavity of the sinus, furnish examples of this condition.

*Diagnosis.*—The diagnosis of dropsies of the maxillary sinus is justified by the presence of a tumor of the os maxillare projecting in one of the aforementioned points; by the yielding nature of the walls of the sinus, which may be depressed and then return upon themselves with a noise like that produced by parchment rubbed between the fingers. But still, in truth, it is possible to mistake an osseous cyst of the maxillary bone for a collection in the sinus; but here the error would be unimportant, since the means employed to cure either of these diseases is equally applicable to the other. The mistake, however, becomes of consequence when dropsy of the sinus is confounded with any other tumor except a cyst. Thus Ferguson had charge of a patient who had been sent to him as affected with disease of the upper jaw, requiring its oblation. The same may be said of the observation of M. Bertrand.‡ Nevertheless, in some exceptional circumstances, surgeons of great experience have mistaken dropsy of the sinus for other diseases of it. Gensoul§ cites an instance. This surgeon having prepared himself to perform resection of the upper jaw, and even taken the first steps in the operation, perceived, as he was about to plunge his instrument into the os maxillare, that he had to do with a dropsy of the cavity of that bone.

* Notes, "Treatise upon the Teeth," by Hunter.

† Bull. de la Societe de la Faculte, t. i, p. 107.

‡ Bull. de la Societe de la Faculte, t. i, p. 107. § Loc. cit.

*Prognosis.*—The prognosis of this disease is not serious. If the affection be left to itself, the distention of the bone will increase, and will be complicated by disturbances created by the compression of surrounding parts; disagreeable enough, it is true, but which in no wise involve the constitution.

*Treatment.*—Dropsy of the antrum being an accumulation of fluid, which tends to increase every day, its treatment offers an indication as positive as rational, which is the evacuation of the contained liquid. This operation may be done through the nasal fossæ, the canine fossæ, the palatine vault, and finally through the alveolar processes. The point of election is that at which the tumor is most conspicuously developed. The evacuation of the fluid through the alveoli is only conventional; it ought to be rejected, unless, indeed, a carious tooth requiring evulsion should offer in its bed a favorable channel for the discharge of the accumulation. The instrument best adapted for the operation is a bistoury or a trocar. With the first, the operation consists in making an incision which is to be kept open for several days; with the second, it consists in a simple puncture. The tumor being pierced, its contents escape, the walls of the sinus collapse, and after a certain time the cavity resumes its normal dimensions. In some instances, however, the opening remains fistulous. Brodie* records an observation of dropsy of the sinus, in which, after being operated upon, the opening remained fistulous for ten years. The observation of Sauve is equally to the point. It was to avoid the inconvenience of a fistula that Blandin had recourse, in one case, to puncture through the alveolus; and he placed in this cavity a pivot-tooth, which enabled the patient to evacuate at pleasure the fluid that accumulated in the maxillary sinus.

* Loc. cit.

[To be continued.]

